# Signal Detection Based on Separable CNN for OTFS Communication Systems

**DOI:** 10.3390/e27080839

**Published:** 2025-08-07

**Authors:** Ying Wang, Zixu Zhang, Hang Li, Tao Zhou, Zhiqun Cheng

**Affiliations:** 1 The School of Electronics and Information, Hangzhou Dianzi University, Hangzhou 310018, China; 90023@hdu.edu.cn (Y.W.); hangli@hdu.edu.cn (H.L.); zhou.tao@hdu.edu.cn (T.Z.); 2 Global Big Data Technologies Centre, University of Technology, Sydney 2007, Australia; zixu.zhang@alumni.uts.edu.au

**Keywords:** orthogonal time frequency space, signal detection, convolutional neural network

## Abstract

This paper proposes a low-complexity signal detection method for orthogonal time frequency space (OTFS) communication systems, based on a separable convolutional neural network (SeCNN), termed SeCNN-OTFS. A novel SeparableBlock architecture is introduced, which integrates residual connections and a channel attention mechanism to enhance feature discrimination and training stability under high Doppler conditions. By decomposing standard convolutions into depthwise and pointwise operations, the model achieves a substantial reduction in computational complexity. To validate its effectiveness, simulations are conducted under a standard OTFS configuration with 64-QAM modulation, comparing the proposed SeCNN-OTFS with conventional CNN-based models and classical linear estimators, such as least squares (LS) and minimum mean square error (MMSE). The results show that SeCNN-OTFS consistently outperforms LS and MMSE, and when the signal-to-noise ratio (SNR) exceeds 12.5 dB, its bit error rate (BER) performance becomes nearly identical to that of 2D-CNN. Notably, SeCNN-OTFS requires only 19% of the parameters compared to 2D-CNN, making it highly suitable for resource-constrained environments such as satellite and IoT communication systems. For scenarios where higher accuracy is required and computational resources are sufficient, the CNN-OTFS model—with conventional convolutional layers replacing the separable convolutional layers—can be adopted as a more precise alternative.

## 1. Introduction

Orthogonal time frequency space (OTFS) modulation [1,2,3,4] has emerged as a promising candidate for the air interface of sixth-generation (6G) wireless networks, particularly in high-mobility scenarios such as vehicular communications, aerial platforms, and satellite systems [5]. Unlike conventional modulation schemes, OTFS transforms the time-varying wireless channel into a two-dimensional (2D) quasi-static representation in the delay–Doppler (DD) domain [6], thereby offering enhanced robustness to both delay spread and Doppler effects. Compared to orthogonal frequency division multiplexing (OFDM), OTFS simplifies signal detection by converting the time-varying channel into a nearly static 2D convolutional model.

In communication environments, multipath propagation and Doppler effects cause significant variations in the time-frequency characteristics of signals. Accurately capturing these variations is crucial to fully harness the performance advantages of OTFS modulation. Although various detection algorithms have been proposed, many of them suffer from high computational complexity, which limits their applicability in practical real-time systems. Consequently, designing reliable yet low-complexity OTFS detection schemes remains a critical challenge for enabling high-performance 6G communications.

Previous studies have shown that linear detectors such as minimum mean square error (MMSE) and linear MMSE exhibit limited performance in highly dynamic environments. In contrast, nonlinear methods, including maximum likelihood (ML), generalized approximate message passing (GAMP), and expectation propagation (EP), improve detection accuracy but incur significant computational costs [7,8,9]. More recently, deep learning techniques have been introduced to OTFS systems, leveraging their strong nonlinear modeling capabilities to enhance detection accuracy while reducing the need for explicit signal detection [10,11].

In this paper, we propose a low-complexity signal detection method for OTFS systems based on separable convolutional neural networks (SeCNN-OTFS). Our work priovides the following key contributions. Firstly, we design a lightweight yet effective feature extraction SeparableBlock structure that integrates global residual learning in the identity path and recursive learning in the residual path, enabling efficient deep feature learning with minimal computational overhead. Secondly, compared to conventional two-dimensional convolutional neural network (2D-CNN)-based detectors, our model achieves an 81% reduction in parameters while maintaining near-optimal detection accuracy, making it highly suitable for resource-constrained systems. Thirdly, the proposed SeCNN-OTFS is particularly advantageous for high-mobility communication scenarios, such as unmanned aerial vehicle (UAV) and vehicle-to-everything (V2X) systems, where computational efficiency is critical.

## 2. Related Work

Research on OTFS signal detection can be broadly categorized into two major approaches: model-driven detection methods and data-driven detection methods. The former relies on physical modeling and probabilistic inference, while the latter leverages data-driven optimization to capture complex transmission characteristics.

### 2.1. Model-Driven Detection Methods

Model-driven techniques include linear detectors, nonlinear iterative detectors, and Turbo-aided structures.

Among the linear approaches, MMSE and zero-forcing (MMSE-ZF) [12] and linear MMSE [13] are widely used due to their low complexity and straightforward implementation. These methods operate in the DD domain and offer initial interference mitigation. MMSE-ZF provides simple equalization, making it suitable for low-mobility scenarios, whereas LMMSE achieves a better performance–complexity trade-off. However, both suffer considerable performance degradation under high Doppler spreads or fast-fading channels.

To enhance detection accuracy, a variety of nonlinear iterative detection algorithms have been proposed. Maximum a posteriori (MAP)–parallel interference cancellation (PIC) [14], message passing (MP) [15], GAMP [7], unitary approximate message passing (UAMP) [16], parallel message passing (PMP) [17], variational Bayesian (VB) [18], and EP [8,9] algorithms introduce Bayesian inference and iterative updates to suppress interference and estimate transmitted symbols more precisely. Some of these methods also integrate domain information or alternate subspace structures for improved robustness. These algorithms often outperform linear detectors, but at the cost of increased computational complexity.

Turbo detection architectures further improve performance by incorporating channel decoding into the iterative process. Turbo maximal ratio combining (MRC) [19] exploits mutual information between detection and decoding stages to refine symbol estimation. Methods like sliding window LMMSE [20] and doubly-iterative sparsified minimum mean square error (DI-S-MMSE) [21] have also been introduced to reduce latency and integrate deep learning enhancements into the iterative MMSE framework. While turbo-aided detection delivers superior bit error rate (BER) performance, it typically incurs significant processing overhead and implementation complexity.

### 2.2. Data-Driven Detection Methods

Deep learning has emerged as a powerful alternative for OTFS signal detection due to its capability to learn complex mappings without explicit channel modeling. These methods utilize large-scale training data to learn the delay–Doppler structure and corresponding symbol representations, making them well-suited to dynamic and non-stationary channel conditions.

Notable architectures include underwater acoustic communication (UWA)-OTFS [22], deep neural networks (DNN) [22,23], 2D-CNN [24], reservoir computing (RC)Net [25], ViterbiNet [26], and OTFS-MatNet [27]. CNN-based models exploit spatial correlation in the DD domain, while RCNet introduces residual connections for training stability. Models like UAM and DNN have been applied to various scenarios including UWA, V2X, and multiple-input multiple-output (MIMO) systems, demonstrating strong generalization across environments. ViterbiNet integrates the classic Viterbi decoding process with learnable neural networks to enable data-driven path selection under low-SNR conditions. 2D-CNN exploits the delay–Doppler channel by learning the MIMO-OTFS input–output relationship. However, the use of 5 × 5 and 7 × 7 convolutional kernels results in increased complexity.

Despite their high accuracy, deep learning-based detectors often involve large parameter sets and intensive computation during inference, limiting their deployment in real-time or embedded systems. As a result, designing lightweight neural architectures, optimizing model compression, and leveraging feature sharing mechanisms are becoming key research directions in this field.

## 3. Problem Formulation

### 3.1. OTFS Transmitter

OTFS modulation maps information symbols onto a 2D DD domain, which can directly capture the geometric features of delay and Doppler shifts caused by relative motion. This mapping transforms time-varying multipath channels into time-invariant channels in the DD domain, making it highly advantageous for high-mobility scenarios.

**Figure 1:**
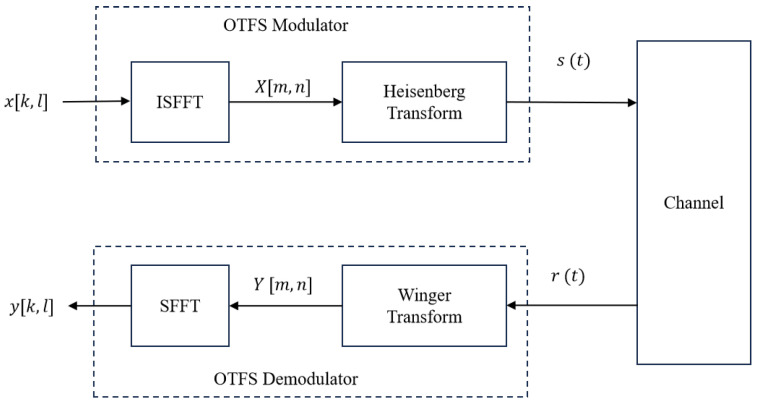
OTFS system.

As illustrated in Figure 1, the OTFS modulator maps the input symbols x[k,l] onto a 2D DD grid, where k=0,1,…,N−1 and l=0,1,…,M−1 represent the Doppler and delay indices, respectively.

The inverse symplectic finite fourier transform (ISFFT) is applied to transform the DD-domain signal into the time–frequency (TF) domain:

(1) $$X\left[m,n\right]=\frac{1}{\sqrt{NM}}\sum_{k=0}^{N-1}\sum_{l=0}^{M-1}x\left[k,l\right]\cdot e^{j2\pi \left(\frac{nk}{N}-\frac{ml}{M}\right)},$$

where X[m,n] is the TF domain symbol at delay index *m* and Doppler index *n*.

Then, the Heisenberg transform converts X[m,n] to the time-domain signal s(t):

(2) $$s\left(t\right)=\sum_{m=0}^{M-1}\sum_{n=0}^{N-1}X\left[m,n\right]\cdot g_{tx}\left(t-nT\right)\cdot e^{j2\pi m\Delta f(t-nT)},$$

where $g_{tx}\left(t\right)$ is the transmit pulse shaping function, *T* is the symbol duration, and Δf is the subcarrier spacing.

### 3.2. Delay–Doppler Domain Channel Model

The signal received after propagation through a time-varying channel is given by:

(3) $$r\left(t\right)=\;\int \int \;h\left(\tau ,\nu \right)\cdot s\left(t-\tau \right)\cdot e^{j2\pi \nu (t-\tau )}\;d\tau \;d\nu +w\left(t\right),$$

where h(τ,ν) is the DD-domain channel response and w(t) is zero-mean complex white Gaussian noise with power $N_{0}$.

In practice, the channel is modeled as a sparse sum of propagation paths:

(4) $$h\left(\tau ,\nu \right)=\sum_{p=1}^{P}h_{p}\delta \left(\tau -\tau_{p}\right)\delta \left(\nu -\nu_{p}\right),$$

where $h_{p}$, $\tau_{p}$, and $\nu_{p}$ are the gain, delay, and Doppler shift of the *p*-th path, respectively.

### 3.3. OTFS Receiver

At the receiver, the Wigner transform maps the received signal r(t) to the TF domain:

(5) $$Y\left[m,n\right]=\int r\left(t\right)\cdot g_{rx}^{⁎}\left(t-nT\right)\cdot e^{-j2\pi m\Delta f(t-nT)}dt,$$

where $g_{rx}\left(t\right)$ is the receive pulse shaping function.

Then, the symplectic finite fourier transform (SFFT) is used to obtain the DD-domain signal:

(6) $$y\left[k,l\right]=\frac{1}{\sqrt{NM}}\sum_{m=0}^{M-1}\sum_{n=0}^{N-1}Y\left[m,n\right]\cdot e^{-j2\pi \left(\frac{nk}{N}-\frac{ml}{M}\right)}.$$

### 3.4. Input-Output Relationship

By vectorizing the DD-domain signals, the overall input–output relationship of the OTFS system can be expressed as:

(7) $$y=H_{\mathrm{DD}}x+w,$$

where x=vec(x[k,l]) is the transmitted symbol vector, y=vec(y[k,l]) is the received symbol vector, $H_{\mathrm{DD}}\in C^{NM\times NM}$ is the DD-domain channel matrix, and $w∼\mathrm{CN}(0,N_{0}I)$ is the vectorized noise.

## 4. Proposed SeCNN-OTFS Based Signal Detection Method

### 4.1. Separable Convolutional Neural Network

Depthwise separable convolution is an efficient convolutional operation that decomposes a standard convolution into two separate steps: depthwise convolution and pointwise convolution. This decomposition significantly reduces computational complexity and the number of trainable parameters.

Depthwise convolution operates independently on each input channel. For an input feature map of size $H_{\mathrm{in}}\times W_{\mathrm{in}}\times C_{\mathrm{in}}$, depthwise convolution applies $C_{\mathrm{in}}$ kernels of size K×K×1, with each kernel convolving over a single channel. The output feature map has a size of $H_{\mathrm{out}}\times W_{\mathrm{out}}\times C_{\mathrm{in}}$. This step extracts spatial features, but does not allow information exchange between channels.

Pointwise convolution then uses $C_{\mathrm{out}}$ kernels of size $1\times 1\times C_{\mathrm{in}}$ to linearly combine the depthwise outputs across channels, projecting them to the desired number of output channels. The size of output feature map becomes $H_{\mathrm{out}}\times W_{\mathrm{out}}\times C_{\mathrm{out}}$. This step enables inter-channel information fusion and dimensionality transformation.

The computational cost of standard convolution is given by the following:

(8) $$O(K^{2}\cdot C_{\mathrm{in}}\cdot C_{\mathrm{out}}\cdot H_{\mathrm{out}}\cdot W_{\mathrm{out}}).$$

The computational cost of depthwise separable convolution includes both depthwise and pointwise parts:

(9) $$O_{\mathrm{depthwise}}=K^{2}\cdot C_{\mathrm{in}}\cdot H_{\mathrm{out}}\cdot W_{\mathrm{out}},\;O_{\mathrm{pointwise}}=C_{\mathrm{in}}\cdot C_{\mathrm{out}}\cdot H_{\mathrm{out}}\cdot W_{\mathrm{out}}.$$

Therefore, the total computational cost of depthwise separable convolution compared to standard convolution is reduced by the ratio of $\frac{1}{C_{\mathrm{out}}}+\frac{1}{K^{2}}$. When K=3 and $C_{\mathrm{out}}=256$, the computational cost is reduced to approximately $\frac{1}{8}$ to $\frac{1}{9}$ of the standard convolution. Similarly, the number of parameters is reduced from $K^{2}\cdot C_{\mathrm{in}}\cdot C_{\mathrm{out}}$ to $C_{\mathrm{in}}\cdot \left(K^{2}+C_{\mathrm{out}}\right)$.

### 4.2. SeparableBlock

The SeparableBlock contains six separable convolutional layers, effectively reducing the computational complexity compared to traditional convolutional layers. The latter three convolutional layers mirror the structure of the first three layers. These tri-layered segments are interconnected through local residual and recursive residual connections, thereby preventing gradient explosion or vanishing gradient issues. The introduction of a channel attention mechanism effectively enhances the model’s ability to identify critical channel features and improves its robustness against noise interference and Doppler spread. This design ensures training stability while maintaining computational efficiency. Furthermore, multiple SeparableBlocks can be stacked in different application scenarios, enabling reliable convergence even as the network depth increases.

### 4.3. SeCNN-OTFS Framework

**Figure 2:**
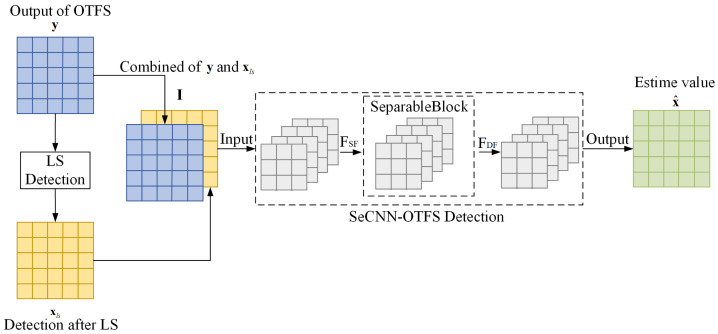
The architecture of SeCNN-OTFS.

**Figure 3:**
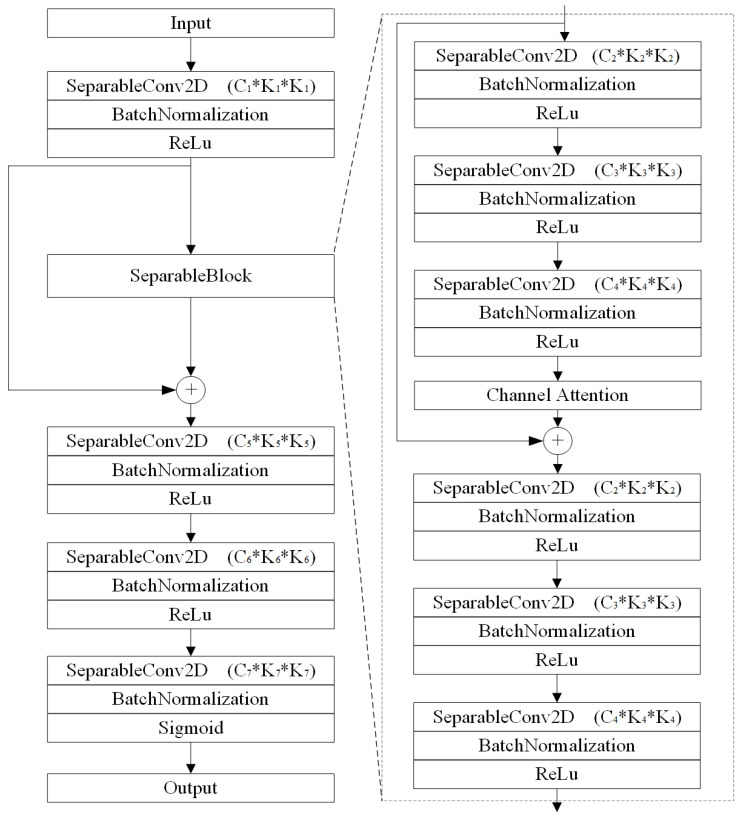
The network of proposed SeCNN-OTFS, including SeparableBlock.

The architecture of SeCNN-OTFS consists of 10 layers of separable convolution, as illustrated in Figure 2. Throughout SeCNN-OTFS, the feature map dimensions remain consistent with the input and output sizes. SeCNN-OTFS primarily comprises three components: shallow feature extraction, deep feature extraction based on SeparableBlock, and reconstruction. A detailed flow of the proposed SeCNN-OTFS network is shown in Figure 3, where the first nine convolutional layers all employ ReLU active function, while the final convolutional layer uses the Sigmoid active function.

Let the input of the network be I, which consists of the output y and $x_{ls}$, given by:

(10) $$I={\left[y^{T},x_{ls}^{T}\right]}^{T},$$

where $x_{ls}$ is the LS estimate of x. As shown in Figure 2, only one SeparableConv2D layer is introduced to extract the shallow feature $F_{SF}$ from the input:

(11) $$F_{SF}=Se_{SF}\left(Input\right),$$

where $Se_{SF}\left(\cdot \right)$ represents the Separable convolution operation.

Then, the extracted shallow feature $F_{SF}$ is utilized for the SeparableBlock to extract the deep feature $F_{DF}$ via:

(12) $$F_{DF}=Se_{DF}\left(F_{SF}\right),$$

where $Se_{DF}\left(\cdot \right)$ denotes the deep feature extraction model based on the SeparableBlock. The SeparableBlock is established to stack three SeparableConv2D layers. Then, the SeparableBlock is connected by the global residual learning in the identity branch and applied using recursive learning in the residual branch.

Finally, the deep feature $F_{DF}$ is mapped into the output by decrementing layer by layer with three SeparableConv2D layers, as follows:

(13) $${\hat{x}}_{m}=F_{SeCNN-OTFS}\left(F_{DF}\right),$$

where ${\hat{x}}_{m}$ is the estimate of x.

This framework extensively uses separable convolutions, significantly reducing the number of parameters and computational cost while maintaining robust feature extraction capabilities. Batch normalization after each Separable convolutional layer stabilizes the training process and accelerates convergence, and ReLU activations provide the necessary non-linearities for capturing complex data patterns. Skip connections in the SeparableBlocks improve gradient flow, ensuring effective training even in deeper network architectures.

Therefore, the loss function of SeCNN-OTFS is defined as the mean square error (MSE) loss:

(14) $$\begin{array}{c} Loss=\underset{{\hat{x}}_{m}}{argmin}||x-{\hat{x}}_{m}{\left|\right|}_{2}^{2}. \end{array}$$

## 5. Complexity Analysis

To quantify the performance of the proposed method, the CNN-OTFS model was used for contrasting the experimental results. It is noted that to make a fair comparison, the network structure of this CNN model is equivalent to that of SeCNN-OTFS, which only replaces the separable convolution with traditional convolution.

To analyze the complexity of SeCNN-OTFS and CNN-OTFS, both the number of parameters and the computational cost are considered in this paper. The computational complexity of convolutional layer is given by:

(15) $$\begin{array}{c} O(K\times K\times C_{\mathrm{in}}\times C_{\mathrm{out}}\times H\times W), \end{array}$$

where K×K is the filter size, $C_{\mathrm{in}}$ is the number of input channels, $C_{\mathrm{out}}$ is the number of output channels, *H* and *W* are the height and width of the feature map. Separable convolution decomposes a convolution into depthwise convolution and pointwise convolution. The corresponding computational complexity is:

(16) $$\begin{array}{c} O(K\times K\times C_{\mathrm{in}}\times H\times W), \end{array}$$

and

(17) $$\begin{array}{c} O(C_{\mathrm{in}}\times C_{\mathrm{out}}\times H\times W), \end{array}$$

respectively. Thus, the total computational complexity for SeCNN is:

(18) $$\begin{array}{c} {\mathrm{FLOPs}}_{\mathrm{SeC}\mathrm{NN}-\mathrm{OTFS}}\approx \sum_{\mathrm{layer}}\left(\left(K^{2}+C_{\mathrm{out}}\right)\times C_{\mathrm{in}}\times H\times W\right). \end{array}$$

The total complexity for CNN is given by:

(19) $$\begin{array}{c} {\mathrm{FLOPs}}_{\mathrm{CNN}}\approx \sum_{\mathrm{layer}}\left(K^{2}\times C_{\mathrm{in}}\times C_{\mathrm{out}}\times H\times W\right). \end{array}$$

## 6. Experimental Result

The dataset used in this paper is generated using MATLAB R2022b [24], consisting of 100,000 samples divided into training, validation, and testing at a ratio of 6:3:1. The simulation parameter settings are shown in Table 1. The subcarrier spacing is set to 15 kHz within a 10 MHz bandwidth. To evaluate the BER of the signal detection against 2D-CNN, a range of SNRs between 0 dB and 20 dB is used with a step size of 2.5 dB. The SeCNN-OTFS is trained with a batch size of 2048, where each sample within a batch corresponds to a unique DD domain channel realization. These realizations are independently generated using random path gains and Doppler–delay indices, ensuring high diversity in the training dataset and allowing the network to generalize well across a wide range of channel conditions. The learning rate is set to 0.01 for a total of 2000 training iterations. A stochastic gradient descent (SGD) optimizer is employed to speed up the convergence. The simulation environment is built on Pycharm Community Edition (python 3.6, Tensorflow 2.15.0).

**Figure 4:**
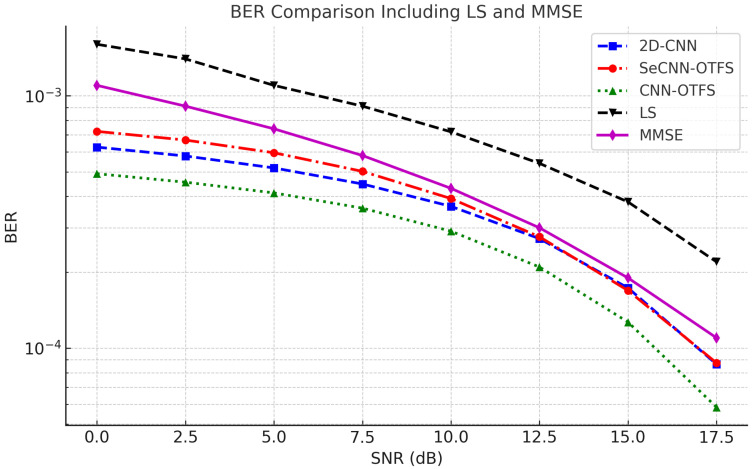
Comparision between SeCNN-OTFS, CNN-OTFS, 2D-CNN, LS, and MMSE of BER.

The parameter configuration of kernel sizes and network layers is summarized in Table 2. All separable convolutional layers use a kernel size of 3×3, and the number of filters gradually decreases in the last three layers, with the final layer containing only one filter to match the output dimensions. To ensure a fair comparison, we construct CNN-OTFS with the same architecture as SeCNN-OTFS, replacing the separable convolutional layers with standard convolutional layers. Similarly, 2D-CNN [24] is included as a baseline model. Also, two classical linear estimation methods of LS and minimum mean square error (MMSE) are introduced as baseline references. The total number of parameters in 2D-CNN, CNN-OTFS, and SeCNN-OTFS are 81,085, 99,501, and 15,511, respectively. Overall, SeCNN-OTFS significantly reduces both computational and parameter cost compared to CNN-based baselines, thereby improving model efficiency and deployment feasibility. The BER performance of different methods under various signal-to-noise ratio (SNR) conditions is shown in Figure 4.

Simulation results indicate that the LS method, due to its lack of consideration for noise statistics, yields the highest BER across all SNR ranges. The MMSE offers a significant improvement over LS in the low and medium SNR regions, but its performance saturates at high SNRs, where the BER cannot be further reduced. In contrast, all three deep learning-based models outperform the classical methods across the full SNR ranges. Notably, 2D-CNN and SeCNN-OTFS share roughly the same BER at high SNRs (over 12.5 dB), while 2D-CNN outperforms SeCNN-OTFS at low SNRs. This is because at high SNR, the parameter efficiency of SeCNN-OTFS is sufficient to learn channel features with less noise, achieving a performance comparable to 2D-CNN. Although SeCNN-OTFS exhibits a slightly inferior BER performance compared to CNN-OTFS across all SNR levels, it achieves a substantial reduction in model complexity. Specifically, the number of parameters in SeCNN-OTFS is only approximately 15% that of CNN-OTFS, and 19% that of 2D-CNN, demonstrating a favorable balance between performance and computational efficiency, as shown in Table 3. Nevertheless, CNN-OTFS may be preferred in scenarios where higher accuracy is required and computational resources permit.

## 7. Conclusions

In this paper, we proposed a low-complexity SeCNN-OTFS method to reduce the computational cost of signal detection in OTFS systems. By replacing conventional convolutional layers with depthwise separable convolutions, the proposed model achieves approximately an 81% reduction in parameter count compared to the 2D-CNN framework. To enhance feature representation while maintaining computational efficiency, we designed a lightweight SeparableBlock architecture, which incorporates global residual learning in the identity branch and recursive learning in the residual branch. Furthermore, a channel attention mechanism was introduced to adaptively highlight salient features while suppressing irrelevant responses, thereby improving robustness under noisy and high-mobility channel conditions. Simulation results confirm that the proposed SeCNN-OTFS achieves competitive BER performance, while significantly reducing model complexity. These findings demonstrate that SeCNN-OTFS is a promising and scalable solution for real-time wireless communication systems, particularly in resource-constrained scenarios such as satellite or IoT communications. 

## Figures and Tables

**Table 1:** Simulation parameter settings.

Parameter	Value
Carrier frequency	4 GHz
Subcarrier spacing	15 KHz
OTFS frame size (N,M)	(8, 8)
Configuration $\left(N_{R}\times N_{T}\right)$	1×1
Modulation scheme	64-QAM
Number of path	P = 4
Maximum speed	500 Kmph

**Table 2:** The parameters setup of the kernel size and layer.

K1	K2	K3	K4	K5	K6	K7
3	3	3	3	3	3	3
C1	C2	C3	C4	C5	C6	C7
32	64	32	32	16	4	1

**Table 3:** Complexity and average BER performance comparison.

Model	Params	FLOPs	Avg. BER	Reduction	Performance Summary
2D-CNN	81,085	115.3 M	3.00 × 10^−4^	Baseline	Best accuracy; high complexity; suitable for powerful devices
CNN-OTFS	99,501	140.2 M	3.83 × 10^−4^	↑ 1.2×	Slightly worse than 2D-CNN; higher complexity
SeCNN-OTFS	15,511	17.8 M	4.27 × 10^−4^	↓ 81%	Acceptable accuracy drop; highly efficient; suitable for embedded systems
MMSE	0	∼0.5 M	9.50 × 10^−4^	N/A	Moderate accuracy; analytical estimator
LS	0	∼0.1 M	1.50 × 10^−3^	N/A	Lowest accuracy; used as benchmark

Note: ↑ indicates increased complexity relative to baseline; ↓ indicates reduced complexity; N/A = Not Applicable (no learnable parameters).

## Data Availability

The dataset in this paper is obtained from Matlab.

## Abbreviations

The following abbreviations are used in this manuscript:

SeCNN	separable oonvolutional neural network
OTFS	orthogonal time frequency space
LS	least squares
MMSE	minimum mean square error
BER	bit error rate
6G	sixth-generation
2D	two-dimensional
DD	delay-doppler
OFDM	orthogonal frequency division multiplexing
ML	maximum likelihood
GAMP	generalized approximate message passing
EP	expectation propagation
SeCNN-OTFS	separable oonvolutional neural network for orthogonal time frequency space
2D-CNN	two-dimensional convolutional neural network
UAV	unmanned aerial vehicle
V2X	vehicle-to-everything
MMSE-ZF	minimum mean square error and zero-forcing
LMMSE	linear minimum mean square error
MAP	maximum a posteriori
PIC	parallel interference cancellation
MP	message passing
UAMP	unitary approximate message passing
PMP	parallel message passing
VB	ariational Bayesian
MRC	bo maximal ratio combining
DI-S-MMSE	doubly-iterative sparsified minimum mean square error
UWA	underwater acoustic communication
DNN	deep neural network
CNN	convolutional neural network
MIMO	and multiple-input multiple-output
ISFFT	inverse symplectic finite fourier transform
TF	e time-frequency
SFFT	symplectic finite fourier transform
MSE	mean square error
SGD	Stochastic gradient descent
SNR	signal-to-noise ratio
